# Editorial: Mucosal immunity to HIV and SARS-CoV-2 infection and vaccination

**DOI:** 10.3389/fimmu.2023.1201650

**Published:** 2023-06-01

**Authors:** Morgane Bomsel, Lucia Lopalco

**Affiliations:** ^1^ Institut Cochin, INSERM, U1016, CNRS UMR8104, Université Paris Cité, Paris, France; ^2^ Laboratory Mucosal Entry of HIV and Mucosal Immunity, Institute Cochin, Paris, France; ^3^ Immunobiology of HIV Unit, San Raffaele Scientific Institute, Milan, Italy

**Keywords:** HIV, SARS-CoV-2, mucosae, immune response, vaccine, IgA

More than 90% of pathogens penetrate the human and animal bodies at mucosal surfaces. In particular, the respiratory and the reproductive tracts have been each targeted by the severe acute respiratory syndrome coronavirus 2 (SARS-CoV-2) and human immunodeficiency virus (HIV), respectively. These two viruses both caused yet not resolved severe pandemics, namely coronavirus disease 2019 (COVID-19) and acquired immunodeficiency syndrome (AIDS).

Although the viral replication cycles and the symptoms these viruses generate differ profoundly, these pathogens share some similarities (1, 2).

SARS-CoV-2 and HIV-1 are both enveloped single strand RNA viruses while their genomic organizations are fundamentally different. Their genetic material is sheltered by a viral membrane in which is embedded an envelope glycoprotein organized as a trimer that is determinant for infection of target cells. The structure and function of the two envelope glycoproteins share similarities although their amino acid sequences entirely differ: both SARS-CoV-2 spike and HIV envelope glycoproteins consist of two subunits, namely S1 and S2 for SARS-CoV-2 and gp120 and gp41 for HIV-1, and function in a similar manner. The surface subunit, namely S1 or gp120, which covers the transmembrane subunit, namely S2 or gp41, allows the attachment of the virus to a specific receptor on the surface of the target cell. This results in a conformational change of the transmembrane subunit, in turn allowing the actual infection of the target cell to take place.

Because of their initial and crucial role in infection, these proteins are attractive antigens to induce protective antibodies, with mitigated success in preventing infection, although in the case of SARS-CoV-2, they have been extremely successful in preventing severe symptoms. Additional vaccine antigens based on internal viral proteins have been considered to activate the cellular immunity, the second arm of the immune response to vaccination.

One of the main obstacles in designing a preventive vaccine based on the viral envelope glycoprotein is the high level of mutations appearing in these two viral envelope proteins during viral replication although mutation rate and mechanisms differs drastically between HIV-1 and SARS-CoV-2. In addition, most vaccines are administered intramuscularly or intradermally and induce an immune response at the systemic level, but not at the mucosal surfaces. Consequently, these classic routes of vaccination show limited ability to prevent the initial infection by viruses such as HIV-1 and SARS-CoV-2 acquired at mucosal site. Therefore, defining the specificity and the role played by mucosal immune responses are timely issues in the fight against the two AIDS and COVID-19 pandemics.

With regard to the immune response to these two pandemic viruses, they share also common aspects (2), which are briefly and not exhaustively described below. Firstly, both viral infections generate an increase in cytokine production. In the case of HIV-1 infection, this increase results in prolonged inflammation (3). In SARS-CoV-2 infection, this cytokine imbalance has suggested therapeutic strategies to block pro-inflammatory cytokines and improve patient recovery (4). Secondly, changes in the gut microbiota have been observed for both infections. In patients infected with SARS-CoV-2 who develop cardiac complications, higher levels of intestinal permeability and activation of inflammasomes are observed (5). In the case of HIV infection, a subset of HIV-positive subjects who are able to control the infection for several years (defined as Elite Controllers) have microbiomes closer to those of healthy individuals than other groups of HIV-infected patients who progress to disease (6). Thirdly, the formation of extracellular neutrophil traps (NETs) is also a common symptom and both viruses can induce a host response known as NETosis (7). NETosis is a process of neutrophil death in which neutrophils release nets of chromatin fibers into the extracellular space. In COVID-19, NETosis is responsible for thrombotic complications, as it is in HIV patients (8, 9).

This Research Topic brings together different aspects of the immune responses to SARS-CoV-2 and HIV during infection and explores how both viruses have targeted, but also subverted, or functionally modulated the host, directly or indirectly, to successfully establish infection.

Pregnancy results in a profound, while physiological, remodeling of female mucosal immunity, including an increase in suppressive regulatory T cells accompanied by a decrease of co-stimulatory molecules. Fenizia et al. investigated whether such immunological adaption can have an impact on the progression of COVID-19 in pregnant women infected with SARS-CoV-2, by altering the early and long-term immunological responses to the virus. During a 6-month follow up, authors elegantly showed that the production of virus-specific antibody was not affected by pregnancy, especially in the infected pregnant women that all developed IgG against the virus. The central memory T cell subset decreased overtime at the cost of an increase in exhausted T effector memory cells. Importantly, by month six postpartum, the inflammatory response caused by COVID-19 was completely resolved. Overall, this study suggests that the maternal immune system was able to mount a protective immune response against SARS-CoV-2 infection while maintaining immunological tolerance toward the foetus.

SARS-CoV-2 uses angiotensin-converting enzyme-2 (ACE2) as main receptor for mucosal cell infection. However, ACE2 and associated proteins of the Renin-angiotensin system (RAS) play an important role in inflammation, immune activation or mucosal barrier maintenance and integrity, as well as in apoptosis. Strikingly, similar ACE2-driven mechanisms contribute also to HIV pathology. Boby et al. raise the important question of the susceptibility of HIV-infected individuals to SARS-CoV-2 infection due to a potential modification of the RAS system. The authors therefore explored a potential modification of ACE2 and RAS-associated protein in a monkey model infected by simian immunodeficiency virus (SIV). Using a transcriptomic approach and expression profiling, they evidenced an alteration of ACE2 and RAS-associated proteins, leading to a disruption of intestinal homeostasis. These modifications could have an impact on acquisition and severity of COVID-19 in HIV infected patients, which needs to be further investigated.

To combat COVID-19, potent vaccines were rapidly developed from the onset of the pandemic, which significantly reduced the severity of COVID-19 outcome. Nevertheless, the response elicited by the vaccine even after two injections is short-lived, so booster doses of the vaccine are needed. Liang et al. questioned the efficacy of a third boost to prevent infection by novel variants of SARS-CoV-2 that emerged over time. In a prospective longitudinal cohort with a follow-up of more than a year, they study in details the anti-SARS-CoV-2 antibodies after a three-dose vaccination. A third dose clearly enhanced rapidly the humoral response, but neutralizing antibodies rapidly declined. It suggests that additional boosters will be necessary to protect the population if the severity of circulating variants persists. This may suggest that the vaccine epitope should be modified to immuno-focus on conserved regions, the mucosal surface should also be targeted to prevent infection and transmission altogether by changing the intramuscular vaccination site with mucosal vaccination (10, 11) to stop the spread of the virus.

As a third partner in the interplay of virus and its host, treatments can also come play and interfere with host immunity. Accordingly, Petkov et al. analysed the impact of pre-exposure prophylaxis (PreP) on HIV-exposed individuals in a randomized controlled trial. They discovered that in young men, short-term PrEP administration of tenofovir and emtricitabine resulted in the systemic production of inflammatory chemokines. These chemokines, including MIP-1beta/CCL4, the natural ligand of the HIV co-receptor CCR5, which is known to block HIV, by impairing HIV envelope gp120 binding to CCR5, may contribute to treatment efficacy. Other chemokines secreted upon PreP administration could serve as biomarkers for potential treatments of side effects. This study paves the way for the use of long-acting formulations and delivery systems for sustained HIV prophylaxis that are currently developed.

Altogether, the contributions to this Research Topic expand our understanding of the interplay between the two mucosally acquired viruses, HIV and SARS-CoV-2, and the host immune system. They open new avenues for the design of infectious disease therapy and emphasize the role of mucosal immunity. Current vaccines widely used in the clinic against SARS-CoV-2 viruses and experimental vaccine formulations tested in preclinical studies for HIV-1 elicit only a systemic immune response with limited efficacy in preventing infection and transmission, in both cases. Therefore, more studies are needed to elaborate mucosal vaccine targeting these surfaces and block the virus at its portal of entry, in addition to elicit a systemic immune response. Moreover, as SARS CoV-2 vaccines are able to drastically reduce the viral burden and severity of the disease, these findings may benefit research of an effective HIV vaccine. Finally, a better understanding of these two pandemic viruses that caused AIDS and COVID-19, would help to anticipate the emergence of novel viremic disease and to develop a treatment to stop their spread.

## Author contributions

MB and LL drafted, revised and edited the editorial and agree to its style and content in its final form.

## References

[1] Fantini J Chahinian H Yahi N. Convergent evolution dynamics of SARS-CoV-2 and HIV surface envelope glycoproteins driven by host cell surface receptors and lipid rafts: lessons for the future. Int J Mol Sci (2023) 24(3):1923. doi: 10.3390/ijms24031923 36768244 PMC9915253

[2] Illanes-Álvarez F Márquez-Ruiz D Márquez-Coello M Cuesta-Sancho S Girón-González JA. Similarities and differences between HIV and SARS-CoV-2. Int J Med Sci (2021) 18(3):846–51. doi: 10.7150/ijms.50133 PMC7797543 33437221

[3] Borges ÁChecktae O’Connor JL Phillips AN Neaton JD Grund B Neuhaus J. Interleukin 6 is a stronger predictor of clinical events than high-sensitivity c-reactive protein or d-dimer during HIV infection. J Infect Dis (2016) 214(3):408–16. doi: 10.1093/infdis/jiw173 PMC4936649 27132283

[4] Giovinazzo G Gerardi C Uberti-Foppa C Lopalco L. Can natural polyphenols help in reducing cytokine storm in COVID-19 patients? Molecules (2020) 25(24):5888. doi: 10.3390/molecules25245888 33322757 PMC7763290

[5] Moccia F Gerbino A Lionetti V Miragoli M Munaron LM Pagliaro P. COVID-19-associated cardiovascular morbidity in older adults: a position paper from the Italian society of cardiovascular researches. Geroscience. (2020) 42(4):1021–49. doi: 10.1007/s11357-020-00198-w PMC7237344 32430627

[6] Vesterbacka J Rivera J Noyan K Parera M Neogi U Calle M. Richer gut microbiota with distinct metabolic profile in HIV infected elite controllers. Sci Rep (2017) 7(1):6269. doi: 10.1038/s41598-017-06675-1 28740260 PMC5524949

[7] Papayannopoulos V. Neutrophil extracellular traps in immunity and disease. Nat Rev Immunol (2018) 18(2):134–47. doi: 10.1038/nri.2017.105 28990587

[8] Saitoh T Komano J Saitoh Y Misawa T Takahama M Kozaki T. Neutrophil extracellular traps mediate a host defense response to human immunodeficiency virus-1. Cell Host Microbe (2012) 12(1):109–16. doi: 10.1016/j.chom.2012.05.015 22817992

[9] Mozzini C Girelli D. The role of neutrophil extracellular traps in covid-19: only an hypothesis or a potential new field of research? Thromb Res (2020) 191:26–7. doi: 10.1016/j.thromres.2020.04.031 PMC7184981 32360977

[10] Russell MW Mestecky J. Mucosal immunity: the missing link in comprehending SARS-CoV-2 infection and transmission. Front Immunol (2022) 13:957107. doi: 10.3389/fimmu.2022.957107 36059541 PMC9428579

[11] Alu A Chen L Lei H Wei Y Tian X Wei X. Intranasal COVID-19 vaccines: from bench to bed. EBioMedicine. (2022) 76:103841. doi: 10.1016/j.ebiom.2022.103841 35085851 PMC8785603

